# Fibrinolysis in Platelet Thrombi

**DOI:** 10.3390/ijms22105135

**Published:** 2021-05-12

**Authors:** Rahim Kanji, Ying X. Gue, Vassilios Memtsas, Diana A. Gorog

**Affiliations:** 1 Faculty of Medicine, National Heart and Lung Institute, Imperial College, London SW3 6LY, UK; rahim.kanji04@imperial.ac.uk; 2 Cardiology Department, East and North Hertfordshire NHS Trust, Stevenage, Hertfordshire SG1 4AB, UK; vassilios.memtsas@nhs.net; 3 Liverpool Centre for Cardiovascular Science, University of Liverpool and Liverpool Heart & Chest Hospital, Liverpool L69 7TX, UK; y.gue@nhs.net; 4 School of Life and Medical Sciences, Postgraduate Medical School, University of Hertfordshire, Hatfield AL10 9AB, UK

**Keywords:** fibrinolysis, thrombin, shear, clot retraction, Factor XIII, clot stability, NETs, platelets

## Abstract

The extent and duration of occlusive thrombus formation following an arterial atherothrombotic plaque disruption may be determined by the effectiveness of endogenous fibrinolysis. The determinants of endogenous fibrinolysis are the subject of much research, and it is now broadly accepted that clot composition as well as the environment in which the thrombus was formed play a significant role. Thrombi with a high platelet content demonstrate significant resistance to fibrinolysis, and this may be attributable to an augmented ability for thrombin generation and the release of fibrinolysis inhibitors, resulting in a fibrin-dense, stable thrombus. Additional platelet activators may augment thrombin generation further, and in the case of coronary stenosis, high shear has been shown to strengthen the attachment of the thrombus to the vessel wall. Neutrophil extracellular traps contribute to fibrinolysis resistance. Additionally, platelet-mediated clot retraction, release of Factor XIII and resultant crosslinking with fibrinolysis inhibitors impart structural stability to the thrombus against dislodgment by flow. Further work is needed in this rapidly evolving field, and efforts to mimic the pathophysiological environment in vitro are essential to further elucidate the mechanism of fibrinolysis resistance and in providing models to assess the effects of pharmacotherapy.

## 1. Introduction

**Figure 1:**
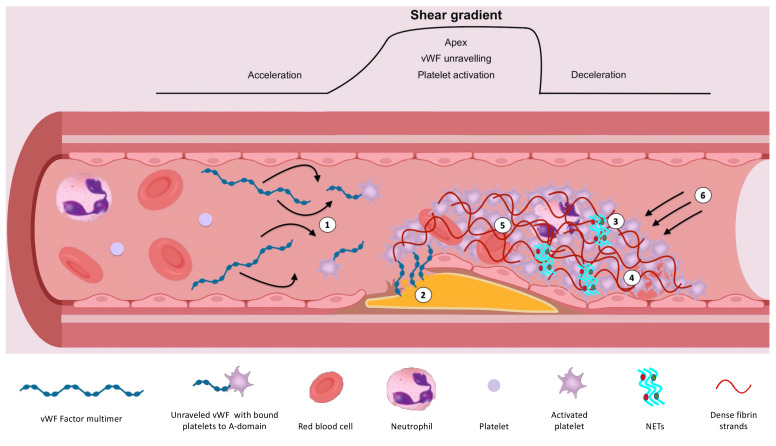
Illustration demonstrating the determinants of endogenous fibrinolysis. (1) High shear/shear gradient results in platelet activation and unravelling of vWF, exposing A-domains for platelet and extracellular matrix binding. This further enhances the affinity of GPIIb/IIIa for fibrinogen. (2) High shear flow contributes to the strength of attachment to the vessel wall. (3) NETs augment coagulation and inhibit fibrinolysis. (4) Platelet-rich clots augment thrombin generation and release of inhibitors including TAFI and PAI-1. This results in densely packed, thin fibrin strands, which are resistant to fibrinolysis. (5) Factor XIIIa-mediated crosslinking of ⍺2-antiplasmin, TAFI and PAI-1 with fibrin inhibits fibrinolysis. (6) Myosin-mediated clot retraction results in increased fibrin density and reduced clot permeability.

Atherothrombotic events are a considerable cause of morbidity and mortality. Much focus and treatment thus far has surrounded the inhibition of platelets due to their crucial role in arterial thrombus formation. However, despite antiplatelet therapy, some patients remain at risk of recurrent thrombotic events. Optimising risk assessment is essential to help identify these patients, with the ultimate aim to reduce events. Emerging data suggest that assessment of endogenous fibrinolysis may help, through identifying patients with impaired endogenous fibrinolysis who are at markedly increased risk of ischaemic events [1,2]. Understanding the determinants of endogenous fibrinolysis is, therefore, paramount, and may highlight new treatment targets (Figure 1).

Interestingly, platelets again seem to be significant modulators of endogenous fibrinolysis, and in this review, we will discuss the mechanisms for mediating resistance to fibrinolysis and the evidence supporting the role of platelets. Areas of discussion will include the role of thrombin, thrombin generation, high shear-induced platelet activation and clot stabilisation, clot retraction, Factor XIII and neutrophil extracellular traps (NETs).

## 2. Role of Thrombin in Endogenous Fibrinolysis

Thrombin is a serine protease that confers significant resistance to endogenous fibrinolysis. It does so through a number of means, which ultimately lead to the formation of a platelet-rich, stable thrombus. Being a potent platelet activator, it maximises the recruitment and aggregation of platelets, which further enhances thrombin release, generating a feed-forward loop. The resultant high thrombin concentration facilitates the formation of a stable clot by cleaving fibrinogen to form insoluble fibrin, which binds platelets together. In addition, it directly inhibits endogenous fibrinolysis through the activation of thrombin activatable fibrinolysis inhibitor (TAFI), which binds and lyses carboxy-terminal lysine residues on fibrin. This prevents the binding and activation of plasminogen to plasmin, which cleaves fibrin into fibrin degradation products [3]. It additionally indirectly inhibits endogenous fibrinolysis through the release of plasminogen activator inhibitor-1 (PAI-1) from platelets, which is a potent inhibitor of tissue-plasminogen activator (t-PA) [4]. The release is activated through intracellular signalling initiated by the g-protein-coupled protease-activated receptor (PAR) [5]. There are two functionally active thrombin receptors found on human platelets, namely PAR-1 and PAR-4 [6,7]. PAR-1 contains a hirudin-like domain possessing a high affinity for thrombin [8], unlike PAR-4, and therefore results in activation at lower concentrations [7]. The activation of these receptors results in the release of the contents of the ⍺-granules of platelets [4], which is responsible for >90% of the circulating PAI-1 detectable during acute arterial thrombosis [9,10].

In addition to this, a number of studies have assessed the relationship between thrombin concentration and fibrin fibre thickness and density [11]. In the presence of a high thrombin concentration, arterial thrombi exhibit thin, densely packed fibrin strands, whilst the converse is seen at lower thrombin concentrations [11,12,13,14,15]. Such structural changes directly impact the resistance of the thrombus endogenous fibrinolysis, as the tiny pores and thin strands impair plasminogen entry and binding with the thrombus, as recently demonstrated in a study of ST-segment elevation myocardial infarction (STEMI) patients using the Global Thrombosis Test and electron microscopy [16]. In this study, impaired endogenous fibrinolysis, assessed in whole blood using a point-of-care technique, showed a correlation with certain structural characteristics of thrombi on electron microscopy, namely reduced fibrin fibre thickness in both the core and periphery of the thrombus and a more densely packed fibrin meshwork, compared to patients with effective endogenous fibrinolysis.

In addition, since the platelet surface plays a central role in the promotion and regulation of thrombin generation, as demonstrated by insignificant generation of thrombin in platelet-poor plasma and a positive correlation observed between platelet number and the extent of thrombin generation [17], platelets are arguably one of the major determinants of endogenous fibrinolysis. This partially explains the difference in resistance to fibrinolysis between arterial (platelet-rich) and venous (erythrocyte-rich) thrombi [18]. Furthermore, the incorporation of red blood cells leads to areas of reduced fibrin fibre density [19].

Understanding the roles of thrombin and platelets is, therefore, key to understanding the determinants of endogenous fibrinolysis.

## 3. Thrombin Generation

After early studies demonstrated the key role of platelets in thrombin generation, subsequent efforts have focused on elucidating the mechanism. Reports of increased exposure of phosphatidylserine from 2% to 12% in an almost on/off phenomenon upon platelet activation [20], and the association seen between the amount of phosphatidylserine on the platelet surface and the extent of thrombin generation [21] indicate that phosphatidylserine is a determinant of thrombin generation. However, the augmentation in thrombin generation is not exclusively related to phosphatidylserine, as it does not replicate the full procoagulant potential of platelets [22]; furthermore, phosphatidylserine has been found on endothelial cells, which are not prothrombotic [23].

More complex platelet interactions and morphological changes are additionally involved, including platelet degranulation with the release of α-granules, platelet ballooning and protein binding. Platelet degranulation in response to thrombin results in the release of coagulation factors, including Factor V, which are activated on the platelet surface [24]. This procoagulant surface is further enhanced as a result of platelet ballooning [25], facilitating greater coagulation factor binding, activation, and assembly of complexes, all amplifying thrombin generation [26]. For those coagulation factors which possess a y-glutamyl carboxyl acid (GIa) domain, (prothrombin, Factor VII, Factor IX and Factor X), PS shows a high affinity [27], whilst other factors such as FVIII are brought close to the platelet surface for thrombin-driven activation through von Willebrand Factor (vWF) binding with the glycoprotein (GP) Ib-IX-V complex [28].

Complexes formed on the platelet surface ultimately increase thrombin generation. This includes the tenase complex, consisting of Factor VIIIa and Factor IXa, which activates Factor X, and the prothrombinase complex made up of Factor Va and Factor Xa, which activates prothrombin [29,30]. Furthermore, in phosphatidylserine-exposed platelets, the above complexes are co-localised, which amplifies their activity 1000-fold [31].

The binding of ligands to platelet glycoproteins further enhances thrombin generation, which is exemplified by GPIIb/IIIa. Upon platelet activation, the affinity of this glycoprotein for fibrinogen increases [32], eventually resulting in a stable thrombus; however, upon binding, outside-in signalling results in further platelet PS exposure and thrombin generation. This, too, is significant, as blocking this receptor with a monoclonal antibody (abciximab) reduces thrombin generation by 40–70% [33,34,35,36].

Clearly, many complex interactions take place; however, the above description may still be an oversimplification. It is now generally accepted that platelet activation and coagulation are not separate processes, and both interplay to generate a stable thrombus by maximising thrombin generation.

## 4. Synergistic Effects of Shear Stress on Platelet Activation

Atherosclerotic plaque rupture, and the resultant exposure of prothrombotic material, including collagen and tissue factor, was previously thought to be the main mechanism behind platelet adhesion, activation and eventual thrombus formation in atherothrombotic events, including myocardial infarction (MI) and stroke. The mechanism behind this includes the binding of GPVI and integrin α_2_β_1_ on the platelet surface to collagen [37,38], resulting in intracellular signalling and platelet activation. As discussed above, this leads to a morphological change in platelets and degranulation, including the release of adenosine diphosphate [39]. This is a potent platelet activator and, through paracrine effects, leads to significant platelet recruitment. It is unsurprising, therefore, that antagonising the P2Y_12_ receptor is the standard of care for patients with ischaemic stroke and MI (in addition to aspirin). 

Plaque erosion is another cause of plaque disruption, which has been shown to account for one-third of acute coronary syndromes [40]. The pathophysiological process differs significantly from that of plaque rupture, and as a result, thrombus content and fibrinolysis potential may differ. Desquamation secondary to degradation of the basement membrane by matrix metalloproteinases [40], and/or apoptosis of the endothelial cells by potent oxidant species produced by myeloperoxidase [41] or shear stress [42], have been proposed. Histological studies have confirmed differences in clot composition, with those formed secondary to plaque erosion being more platelet-rich [43]. This could be attributable to a burst of tissue factor expression by endothelial cells in response to potent reactive oxygen species produced by myeloperoxidase [41], and also by the migration and recruitment of neutrophils secondary to the release of chemokines and endothelial cell injury, with resultant neutrophil extracellular trap (NET) formation [44]. NETs have the ability to acquire tissue factor, platelets and fibrin, facilitating the formation of a platelet-rich thrombus [45,46].

Another important mechanism of arterial thrombosis is shear-induced platelet activation. At low shear levels, vWF circulates as a large multimer [47]. In this conformation, its A-domains, which are required for binding to the platelet and extracellular matrix, are concealed. However, high shear leads to unravelling/uncoiling of vWF [48]; in fact, in the presence of a shear gradient, such as that seen at sites of coronary stenosis, this unravelling occurs with greater efficiency as the proximal and distal ends of the multimer experience differing pulling forces, resulting in unravelling at lower shear [49]. Furthermore, in the presence of an atherosclerotic plaque rupture and growing thrombus, shear forces and gradients will increase further, leading to further unravelling, thus initiating a cycle.

This unravelling and elongation exposes the A1 domain, which binds to the platelet GPIbα receptor, leading to platelet adhesion and subsequent aggregation [50,51]. This occurs in addition to the aggregation driven by the binding of fibrinogen to GPIIb/IIIa, with the two processes therefore working in synergy to form a stable thrombus. In fact, recent evidence suggests that the binding of GPIbα with vWF increases the affinity of GPIIb/IIIa for fibrinogen [52]; thus, it enhances platelet aggregation. 

The role of platelet activation under high shear flow conditions, causing platelet aggregation and being a determinant of fibrinolysis, is supported by recent data from the RISK-PPCI study, in which endogenous fibrinolysis was assessed using the Global Thrombosis Test in patients presenting with STEMI [1]. In these patients, time to form an occlusive thrombus under high shear conditions in vitro correlated inversely with the effectiveness of endogenous fibrinolysis, implying that shear-activated platelets contribute to impaired endogenous fibrinolysis.

This is very relevant, as current pharmacotherapy for patients with arterial thrombosis is directed mainly at antagonising the P2Y_12_ receptor and inhibiting cyclo-oxygenase, which have no effect on this shear-driven, vWF-dependent pathway of platelet aggregation and activation. Furthermore, inhibition of the P2Y_12_ receptor has not been shown to affect endogenous fibrinolysis [53].

Additionally, this shear-driven mechanism for aggregation is not dependent upon plaque rupture, and so patients with significant stenoses are at risk of both thrombus formation and impaired endogenous fibrinolysis. Furthermore, this risk may be dependent on the degree of stenosis and plaque burden. With increasing luminal narrowing, the shear gradient increases, resulting in increasing platelet activation at the apex of the lesion. Studies have demonstrated platelet activation [54], including microparticle formation in response to shear [55], and increased activation secondary to platelet hammering (exposure to repeated hyper-shear) [56]. Additionally, increased phosphatidylserine externalisation and procoagulant activity, including thrombin generation, have been observed at high shear rates [57], which appear to be dependent upon the binding of vWF and the GPIbα platelet receptor [58,59]. This receptor has a mechanosensitive domain, which unfolds when bound to the A1 domain of vWF, leading to intracellular signalling [60] and intermediary activation of other integrins, increasing their affinity for ligand and facilitation of outside-in signalling [60]. Furthermore, since GPIbα can also bind soluble vWF [61], and since platelets remain sensitised after exposure to high shear [62], hyper-aggregation can be seen downstream from the site of maximal luminal stenosis [63], where deceleration and low shear favour thrombus formation.

Studies aiming to block this high shear-driven platelet aggregation using monoclonal antibodies to the A1 domain of vWF have shown reduced thrombin generation, adhesion and aggregation [64]. Furthermore, they have shown reduced bleeding times when compared with abciximab [65], highlighting specificity for shear-driven activation and therefore allowing aggregation of platelets at low shear with fibrinogen and GPIIb/IIIa. However, whether this formally affects endogenous fibrinolysis is unclear.

## 5. Clot Retraction

Clot retraction is a physiological mechanism to aid healing during haemostasis. Through the expulsion of serum, which has been depleted of clotting factors, the volume of the clot reduces, leading to the coming-together of wound edges [66]. It therefore has favourable effects in physiology and is platelet-mediated. After the binding of fibrinogen to the GPIIb/IIIa receptor, phosphorylation of the receptor leads to outside-in signalling, resulting in myosin binding [67] and co-localisation of the ANK domain containing Bcl-3 with the cytoskeleton, which is tyrosine kinase dependent [68].

Thus, when contractile forces are generated by myosin [69], this leads to clot retraction through its connection with the GPIIb/IIIa receptor and fibrin(ogen), which is crosslinked with other fibrin strands and platelets [70]. Therefore, the greater the platelet number and fibrin crosslinking are, the greater the force and effects of clot retraction are. This has the overall effect of increasing fibrin density and reducing clot permeability [71], which, as mentioned above, affects fibrinolysis. Thin, densely packed fibrin strands are resistant to fibrinolysis, and reduced permeability and pore size impairs the entry of plasminogen and t-PA [72].

This effect on fibrinolysis has been shown both in vitro and in vivo. In a mouse model of thrombus generation through mesenteric vein injury and thrombin injection, clot retraction was seen to occur over a period of 3 h, which was inhibited by blebbistatin, a potent myosin IIa inhibitor [69]. Furthermore, when recombinant t-PA was infused over the thrombi, the lysis of unretracted thrombi was far greater than that of retracted thrombi; in fact, a relationship was seen between the degree of lysis and clot retraction. However, what was unexpected was the potential role of early limited endogenous fibrinolysis in clot retraction. When t-PA was infused early following clot formation, fibrinolysis was seen with a reduction in both thrombus volume and fibrin. However, after 30 min, thrombus volume reduced, with no effect on fibrin. Furthermore, pre-treatment of mice with tranexamic acid, an inhibitor of fibrinolysis, led to impairment of early clot retraction. This effect was confirmed in vitro, where low concentrations of t-PA, in fact, facilitated clot retraction; however, at higher doses, lysis was observed.

These findings have been replicated somewhat using human blood in vitro [73]. Retracted clots were found to be resistant to external fibrinolysis; however, this was not the case for endogenous fibrinolysis. For retracted clots that had been bathed in t-PA prior to their formation with thrombin, the rate of endogenous fibrinolysis was higher when compared with clots that had been prepared in a similar manner but with the addition of inhibitors of retraction.

Clearly a link exists between platelet-mediated clot retraction and endogenous fibrinolysis.

## 6. Factor XIIIa

Factor XIII is a coagulation factor that has many functions, primarily directed at promoting clot stability. Its timely release from activated platelets with fibrinogen, prothrombin, Factor V and Factor VIII (Table 1), all required during the later stages of clot formation, ensures its abundance when required to stabilise the formed thrombus. It can also be found in plasma complexed with fibrinogen, where it is activated by thrombin.

One of its many stabilising effects includes the crosslinking of fibrin [74], which is particularly important in environments of high shear [75]. Furthermore, through promoting coupling with protofibrils, deformation at low shear is prevented through stiffening [76]. Coupling also has the effect of reducing the size of the pores and impairing the entry and diffusion of fibrinolytic enzymes, including t-PA, into the clot [77].

Factor XIIIa also facilitates the crosslinking of α2-antiplasmin [78], TAFI and PAI-1 with fibrin. Crosslinked α2-antiplasmin prevents adsorption of plasminogen with fibrin, preventing its activation and lysis of fibrin. Furthermore, in plasma, it binds and inhibits plasmin. Therefore, it has a central role in inhibiting endogenous fibrinolysis.

However, in vivo studies representing its effects on endogenous fibrinolysis are limited. Reed et al. undertook a study involving anaesthetised ferrets with pulmonary embolism and found significantly enhanced endogenous fibrinolysis activity in ferrets treated with a Factor XIIIa inhibitor [79]. Furthermore, total Factor XIIIa inhibition resulted in greater endogenous fibrinolytic activity compared with only α2-antiplasmin-inhibited crosslinking, suggesting that Factor XIIIa-mediated fibrin crosslinking also plays a major role in endogenous fibrinolysis.

In vitro human studies are greater in number and confirm the role of Factor XIII in fibrinolysis. In a study by Jansen et al., t-PA was added to fresh human whole blood prior to clot formation with the addition of thrombin [80]. Fibrinolysis was greater in the samples that had antibody-inhibited Factor XIIIa activity. These findings were reproduced in a study using plasma clots and a Chandler loop [81]. Interestingly, they both also concluded that the majority of the inhibitory effect of Factor XIIIa on fibrinolysis was mediated through α2-antiplasmin.

## 7. Activated Neutrophils and NETs

Neutrophil extracellular traps (NETs) are web-like structures composed of DNA and histones [82]. They are released by activated neutrophils, in addition to elastases, and have a significant effect on coagulation. Histones specifically activate platelets [83], inhibit activated protein C-mediated inhibition of coagulation [84] and support thrombin activation [85]. DNA can activate Factor XII and initiate coagulation [86], whilst elastases can break down inhibitors of coagulation [87].

There is now evolving evidence that further highlights the effect of NETs on fibrinolysis. One group reported on the effects of histone–DNA complexes, which resulted in the formation of thrombi with reduced permeability, in both fibrin [88] and plasma clots [89], and prolongation of t-PA-mediated fibrinolysis. This effect was reproduced when activated neutrophils themselves were added to plasma (with confirmation of NET formation using electron microscopy) and reversed with the addition of DNAse, implicating a contributory role for DNA (in NETs) in inhibiting fibrinolysis. Further evidence suggests that elastases bound to DNA in NETs are responsible for plasminogen degradation, and this may be one mechanism behind fibrinolysis resistance [90].

Further human data are limited to ex vivo and in vitro studies. A histological analysis of clots retrieved from 108 patients undergoing endovascular therapy for acute ischaemic stroke confirmed the presence of NETs, which correlated with procedure time [91]. When the retrieved clots were then treated with t-PA, the administration of DNAse hastened lysis time. In 126 patients treated in hospital for pulmonary embolism, raised lactate levels were associated with a 29% higher neutrophil count, 45% higher plasma citrullinated histone H3 level, reduced plasma fibrin clot permeability and longer clot lysis time [92]. Furthermore, lactate positively correlated with plasma citrullinated histone H3 concentration, plasma clot lysis time and PAI-1 level.

Thus, NETs confer resistance to endogenous fibrinolysis. The mechanism may be multifactorial and includes the protection of thrombin from degradation (and resultant dense fibrin clot formation) and the promotion of plasminogen breakdown by bound elastases. 

## 8. Clot Stability

Clot stability refers to the ability of a thrombus to resist fibrinolysis and dislodgement from the vessel wall by flowing blood. The former has been discussed extensively within this review, but the mechanisms determining the strength of attachment to the vessel wall have not been addressed. The latter appears to be mediated through shear stress. With increasing wall shear, an increasing number of platelets are recruited to the growing thrombus [93]. Under high wall shear conditions, the formed thrombus has a thicker shell and a more densely packed core [94]. This may be facilitated by the shell preventing washout of platelet activators, thus promoting paracrine activity [95], with the resultant thrombus being resistant to fibrinolysis. Furthermore, the strength of attachment to the vessel wall is increased by high shear flow, and this may be secondary to the high affinity state of the A1 domain of vWF for GPIbα under these conditions [50]. However, a point is reached where the risk of dislodgement is greater; furthermore, Shi et al. suggest that wall shear may also have a contributory role, demonstrating a parabolic relationship between wall shear and thrombus area [94].

Clearly, increasing wall shear stress and shear flow play a role in clot stability; however, their effects are not linear. A point is reached where the bond with the vessel wall is overcome, leading to thrombus dislodgment. This may have an effect on endogenous fibrinolysis potential, as microemboli may have exposed areas for the entry of fibrinolytic enzymes, resulting in more rapid fibrinolysis than the original mother thrombus.

ADP signalling also appears to be involved in clot stability. Administration of P2Y_12_ inhibitors to whole blood has been shown to destabilise thrombus formation under high shear in vitro, resulting in microbleeds [53]. The effect was more profound with more potent inhibitors such as cangrelor, and this also enhanced endogenous fibrinolysis.

Furthermore, in an in vitro study, the administration of ticagrelor after the initiation of clot formation through the exposure of ADP and collagen led to dispersion, confirmed by aggregometry [96]. This has also been shown in vivo in a murine model, where early arterial thrombotic occlusion was partially reversed with the administration of ticagrelor.

Factors that potentiate clot stability may, therefore, confer resistance to endogenous fibrinolysis.

## 9. Conclusions

In conclusion, there are many mechanisms involved in controlling endogenous fibrinolysis. There is a significant contributory and complex role of cellular components, particularly platelets and NETs, in determining resistance to endogenous fibrinolysis. Additionally, high shear flow conditions further impact platelet activation and thrombus stability. Further work is needed in this rapidly evolving field, and efforts to mimic the pathophysiological environment in vitro are essential to further elucidate the mechanism of fibrinolysis resistance and in providing models to assess the effects of pharmacotherapy.

## Figures and Tables

**Table 1:** Site of synthesis and main source of major pro- and anti-fibrinolytic proteins discussed within this review.

Protein	Origin	Major Source
Factor XIII	Cellular (megakaryocytes, platelets, monocytes, osteoblasts) Plasma	Platelets
Factor V	Liver, platelets and plasma	Liver
Factor X	Liver and plasma	Liver
Factor VIII	Liver, endothelial cells and plasma	Liver
Prothrombin	Liver and plasma	Liver
Plasminogen activator inhibitor (PAI)	Endothelial cells, liver, adipose tissue, plasma and platelets	Platelets
Tissue plasminogen activator (t-PA)	Endothelial cells, mesothelial cells, megakaryocytes and plasma	Endothelial cells
Fibrinogen	Liver, plasma (predominantly), platelets, lymph and interstitial fluid	Liver
⍺2-antiplasmin	Liver and plasma	Liver

## Data Availability

Not applicable.

## Abbreviations

GP	Glycoprotein
GTT	Global Thrombosis Test
MI	Myocardial infarction
NETs	Neutrophil extracellular traps
PAI-1	Plasminogen activator inhibitor-1
PAR	Protease-activated receptor
PS	Phosphatidylserine
STEMI	ST-segment elevation myocardial infarction
TAFI	Thrombin activatable fibrinolysis inhibitor
t-PA	Tissue-plasminogen activator
vWF	von Willebrand Factor
